# Systematic observation of participatory interaction in university lectures: a multiple case study with a mixed methods approach

**DOI:** 10.3389/fpsyg.2024.1410486

**Published:** 2024-11-01

**Authors:** Héctor Tronchoni, Conrad Izquierdo, M. Teresa Anguera

**Affiliations:** ^1^Faculty of Teacher Training, University of Valencia, Valencia, Spain; ^2^Faculty of Psychology, Autonomous University of Barcelona, Barcelona, Spain; ^3^Faculty of Psychology, Institute of Neurosciences, University of Barcelona, Barcelona, Spain

**Keywords:** participatory interaction, mixed methods, multiple case study, systematic observation, university lectures

## Abstract

**Introduction:**

In order to improve and innovate the face-to-face instructional task in postgraduate and doctoral university teaching encompasses different formats of participatory interaction (PI), considered to be social medium facilitators of deep learning, including the development of autonomous expert activity. The purpose of this article is to establish the use of systematic observation and lag sequential analysis as a conceptual-methodological choice to base the review of social-constructivist instructional practice that is taught in an expository format prepared by the teacher.

**Method:**

The systematic observation of the expert’s expository session from its inception to its conclusion was approached from a mixed methods perspective as a subject of multiple case studies. A total of four postgraduate teachers were selected. A purpose-built observational instrument was constructed. The data quality was evaluated with intra-observer agreement tests by calculating Cohen’s kappa coefficient (k). After the data matrices for each case were obtained, all possible file aggregations of the data were performed to detect the existence of common structures in the patterns through lag sequential analysis.

**Results:**

The sequential patterns of replicated and common lags of the multiple cases describe the chaining of the observed events, which characterizes the participatory interaction. Twelve lag sequential patterns have been identified that function as dialogical links, generated by the probability that the category “question” is linked to the conditioned events of “speech direction” and “exchange orientation.”

**Discussion:**

Having constructed a theoretical interpretative scheme of the replicated patterns, we discuss the results. First, the significant results of the lag sequential analysis as examples of basic patterns extracted from their way of conducting expert expository sessions. As such, they can be reviewed with the formative purpose of reflecting on their potential for change when they are understood as dialogical links of participatory interaction committed to deep learning and the development of expert autonomy. Second, there is a training step consisting of the use of self-observation and the observation that teachers can make of the expert expository task. Finally, we conclude that non-intrusive systematic observation is a good choice when channeling the gradual and renewed improvement of participatory interaction with an expert expository format (§EF) and a mixed methods methodology.

## 1 Introduction

Despite not overlooking the issue of the conceptualization and measurement of student learning throughout academic education in the countries of Organisation for Economic Co-operation and Development (2008), it is of great interest to education centers and their professional teams to come up with novel ideas to help create educational experiences that provoke deep or expert learning (Hattie, 2015; Houser and Hosek, 2018; Stobart, 2014).

This article, which complements previous publications on instructional communication by employing an expert expository format derived from the university lecture (Tronchoni et al., 2018b; Tronchoni, 2019; Tronchoni et al., 2021, 2022), falls within the chain of joint efforts made by an education center and its teaching staff to enhance their teaching practice, firmly committed to an active social-constructivist vision of the learning process (Chi, 2009).

Within the framework of participatory action research (Elliot, 1991), our research commitment with the center and participating lecturers consisted of analyzing how the teachers—in their interactions with the attending students—gave expert expository lessons. These lessons were designed as a mode of participatory interaction (PI) based on a kind of listening (Barker, 1971; Rogers, 1984) that entails the recognition of the dialogic experience (Bakhtin, 1981) of the overlapping, nested, crossed, or defined alternation of speaking-speaking turns, together with confirmatory sympathy (Watzlawick and Weakland, 1977), and the sharing of the active-comprehensive state of the interlocution (Pérez Fernández, 2008; Van Dijk and Kintsch, 1983) initiated, maintained, and closed by the expert format.

The research-educational focus of the improvement of participatory interaction with an expert expository format (from now on PI§EF) was designed in accordance with the possibilities offered by observational methodology within the framework of the mixed methods paradigm (Bazeley, 2018). The challenge of integrating qualitative and quantitative processes in data extraction and analysis is inherent in non-intrusive observational methodologies, preferably based on systematic methods of diachronic analysis of the continuous stream of behavior events in context (Alvarado-Álvarez et al., 2021). The categories to be observed and coded for later quantitative and qualitative analysis must be well defined and accessible to external observers in order for the study of them to be replicated (Anguera et al., 2021; Izquierdo and Anguera, 2021).

It should be noted that the systematic framework of observation—conceived as a specific way to access the quantification of behavior—is distinguished from other quantitative methodological options present in psychological research in that it incorporates, controls, and verifies the shared criteria of scientific rigor and its connections with the objective of the study. Therefore, it is imperative to consider the sensitivity and conditions that impact the process of obtaining, measuring, and analyzing situated behavior, which must be taken into account, preferably from a sequential perspective (Bakeman and Gottman, 1986). In the Method section, the necessary indications are incorporated step by step to enable understanding of how the research was conducted and to be able to offer a sufficient degree of precision and replicability, although it is not without limitations (*Vide*, Section 4).

Thus, in the methodological context of systematic observation, we carried out a multiple case study in accordance with the case aggregation procedure (Anguera, 2018) that incorporates the suggestions of Stake (2006) and Yin (2014). Our objective of conducting an intensive inquiry focuses on the momentary and dynamic aspects of PI§EF, with the idiographic aim of exploring the existence of patterned regularities in the multimodal instructional processes shared by the participants (*Vide*, Section 2.3).

The educational task of teaching and supporting learning (Wertsch, 1988) implies guiding the process of content acquisition via student-teacher interaction in order to achieve the goals of the activity (Van Dijk et al., 2020). In the expert expository task, the instructional communication pivots between “giving” information about what is known and “asking questions”^1^ about how it is known. The presence or absence in the participants—total or partial—of this two-fold game of intentions will leave a mark on both verbal and non-verbal modes of activating the lecturer’s expository plan from both extremes of the interaction. In effect, both actions foster the planning and execution of the lecturer’s explanation, and both actions connect with the constructive activity of the students’ active-comprehending listening (Pérez Fernández, 2008; Van Dijk and Kintsch, 1983). It can be inferred that the educational objectives put into circulation by “give” and “question” are spontaneously shared and anticipated by the mere act of executing out the assigned task in the classroom (Goffman, 1974; Poyatos, 1983). It is imperative to be adequately prepared for the PI§EF challenge, given the potential risks and obstacles that may result in a demoralizing outcome.

Our research focuses on the systematic observation of PI§EF on the cognitive and socio-affective processes of active-comprehending listening, which is stimulated by the acts of speaking and discursive and semiotic strategies (Coll and Onrubia, 2001; Sperber and Wilson, 2004; Van Dijk, 1985, 2000; Van Dijk and Kintsch, 1983). This dialogued expository mode is expressed in the verbal and non-verbal behavior of the observable exchanges within the expert’s speaking turn (Duncan, 1973; McCarthy, 2003) and, to a lesser extent, in the rotation between turns (Duncan and Fiske, 1977; Poyatos, 1983).

In accordance with our conceptualization (Tronchoni et al., 2018b), the invitation to dialogical cooperation stemming from active-comprehending listening implies something more than mere factual recognition (Kilby, 2021) of the acknowledgement of the information provided at each point in time. The communicating actors observe, listen, write, ask, answer, etc., and these observable actions can be interpreted within the framework of the declared intentions plan and/or performed by the participants as the tip of the iceberg of the non-mechanical deep processing (Ausubel et al., 1978; Bruner, 1960) of interactive minds (Staudinger and Baltes, 1996). These externalize the internal dialogue manifested in the *auditor back channels* activities (Duncan, 1973) and in the brief alternate couplings—secondary alternating, alternation filling in, or overlapping—between the students and the teacher, employing verbal and non-verbal language that is almost like shorthand and unique to the group to communicate what is being understood and, therefore, consequently shared (Schön, 1987).

Our vision of the role played by PI§EF is rooted in the educational principles of social-constructivist psychological development (Coll and Onrubia, 2001; Wertsch, 1988); in addition, we are also concerned about universal design for learning (UDL) principles (Mottet, 2015; Novak, 2016). When we talk about the construction of expert knowledge, we refer to the socio-affective/cognitive learning changes and the micro changes or small steps that the actors experience and express as dispositional movements and/or instrumental tools. These changes contribute to the growth of autonomous expertise. We have identified the involved mechanisms of educational support as fluid or sequential scaffolding (Bruner, 1978), depending on the contribution of both extremes in the interaction, and as teacher-planned scaffolding aided by high or low technology (Yelland and Masters, 2007). In the context of co-constructive (Monereo, 2009) step-by-step improvement, diachronic systematic observation provides us with consistent lag sequential analysis results, enabling us to advance—in our case—in the formation of fluid scaffolding and the preparation and execution of planned scaffolding.

To be more precise, and by way of summary, so far we have referred to: (a) the fluid otherness sustained by the motives and baggage of previous experiences and knowledge that nurture cooperation in the organization of active-comprehending listening shared by both extremes of the interaction; and (b) the collaboration via the use of strategies and abilities linked to the problems anticipated by the teacher and those that arise that shake the socio-cognitive/emotional adjustments of the PI§EF (Tronchoni et al., 2021).

The scientific and professional interest that this approach to PI§EF—based on expert learning with global and dynamic social-constructivist modeling—might have for university lecturers (Mandl et al., 1996) has been subjected to a process of systematic review (Tronchoni et al., 2022).

The results obtained are unfavorable, despite the fact that the framework of systematic evidence employed verified the scientific visibility of the existence of a determination to renew the expository lecture format prevalent across different continents. This is supported by higher education institutions and educational research centers, and it is recognized and demanded by a wide range of existing fields of knowledge. The suggestion for this renovation is limited to Incorporating ICTs (Information and Communication Technologies) and the use of strategies, techniques, and practices that foster motivation, commitment, and active learning. These are certainly improvements, but they are also at risk of reducing the expert expository format to a mere anecdote since no value is placed on the dialogued condition of active-comprehending listening.

Hence, it can be asserted that despite having established the existence of a widespread interest among universities over the five continents in transforming the expository format inherited from the lecture class, no specific formative treatment has been identified for the comprehensive, in-depth development of the principle of activity linked to the processes of construction and interaction of learning (Chi, 2009) with an expert expository format (§EF). This vacuum is what has driven us to develop and obtain evidence about PI§EF.

Finally, we conclude this section by establishing the prerequisites of the expert expository format and furnishing PI§EF with an operational definition. The §EF rules that we have taken into consideration are: *R1*, the rule of shared commitment between both extremes of the interaction, with active-comprehending listening; *R2*, the rule of “give-receive/receive-give” in accordance with the expert’s fundamental actions of give/question; and *R3*, the rule of the asymmetric constriction of the speaking time in the students’ turns.

**Figure 1:**
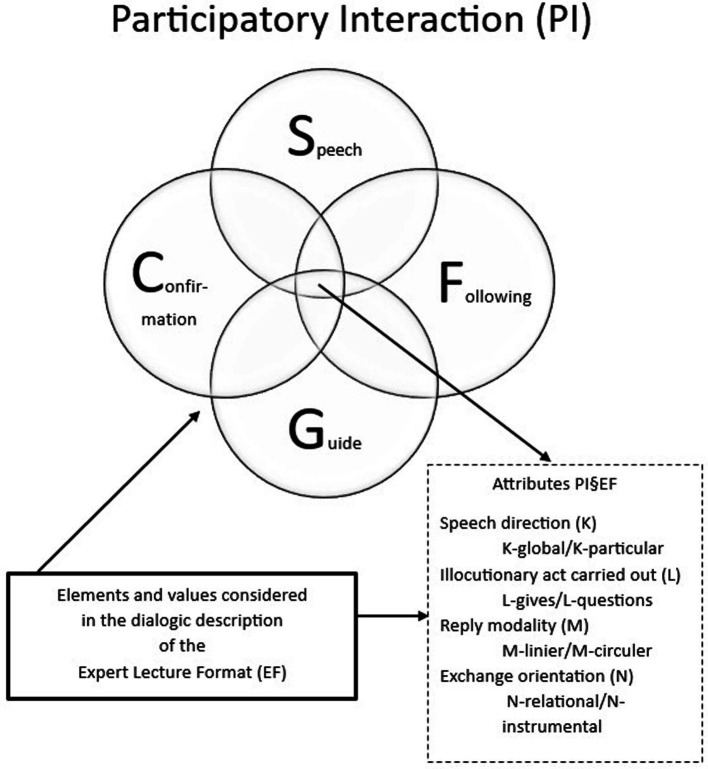
Representation of the intersectional relationship (∩) between the four structural components of Participatory Interaction (PI§EF) and the values considered for the attributes of the expert expository format in the framework of the diachronic observational study carried out. Own production.

We conceive the expert expository moment—independently of the duration of the explanation—as a PI experience lived and relayed by the listeners with these words: *speak, speak, and do not stop* (S), *because we follow you* (F) *and you understand us* (C) *and also follow us* (G)*… In this way, together we will learn to be experts* (PI§EF).^2^ By using the algebra of sets, we have:


PI§EF=S∩F∩C∩G

where, PI§EF = x/x ∈ S ^ x ∈ F ^ x ∈ C ^ x ∈ G. In accordance with this expression, we define participatory interaction (PI), made up of the rules of the expert expository format (§EF), as a set of values assigned to the properties or attributes common to the four components: (1) *the teacher speaks* (S), (2) *the students follow* (F), (3) the actors feel the *confirmation of the relationship* (C), and (4) the actors share *the help that guides the learning* (G). The intersection of the four components encompasses the following attributes: the direction of the speech (K), the illocutionary action of the intervention (L), the modality of the response (M), and the orientation of the exchange (N) (*Vide*; Figure 1).

Finally, from the point of view of union (∪), the disjunctive attributes (˅) include the specific and distinctive elements of each component in accordance with the situations covered (*Vide*, Section 2.2).

## 2 Method

The existence of systematic observation in the disciplinary field of analysis of social interaction involving multimodal human communication is due to important methodological advances tied to conceptual, statistical, and computer aspects (Anguera and Izquierdo, 2006; Sackett, 1980).

In this section, we incorporate, on the one hand, the advantage of having bridged the gap between the qualitative and the quantitative by allowing the observational records of interactive behavior—that are categorical in nature—to undergo a quantitative treatment through the construction of an organized data matrix in accordance with certain parameters. This is made possible through the use of novel robust techniques of quantitative analysis applicable to social interaction (Anguera et al., 2021).

On the other hand, the incorporation of a multiple case study as a methodological aim (Anguera, 2018) has allowed us to go deeper into the proposed observational problem without losing intensity; i.e., lag sequential analysis (Sackett, 1980) applied to the data matrix of each lecturer participating in this research (n = A, B, C, and D) is completed by verifying the existence of identical structures in the behavioral patterns detected via the aggregation of data from the four participating lecturers: A, B, C, and D; AB, AC, AD, BC, BD, and CD; ABC, ABD, ACD; BCD; and ABCD (Anguera, 2018; Yin, 2014).

### 2.1 Design

An applied observational design is N-I/P/M (Anguera et al., 2001). The study is characterized as nomothetic on a primary level of analysis due to the presence of four lecturers, idiographic on a secondary level due to the presence of multiple cases, punctual with intra-sessional follow-up due to the recording of a single session per teacher from the start to the finish of the class, and multidimensional due to its theoretical framework and its structure in two macro dimensions.

This study conforms to the characteristics that establish the profile of observational methodology: (1) the study of spontaneous and perceptible behavior is studied, in the usual contexts where it occurs; (2) the subsequent stages that follow on from the establishment of the design consist of the construction of an *ad hoc* instrument, computerized registration, data quality control, and data analysis; (3) the custom-built instrument is non-standard, and its laborious preparation necessitates the proposal of certain dimensions based on the theoretical framework and reality that can be deployed in sub-dimensions, each of which produces a comprehensive and mutually exclusive system of categories; (4) the computerized record is tailored to the categorical nature of the data; (5) data quality control ensures the agreement of the records of different observers (inter-observer) or of the same observer at different moments in time (intra-observer); and (6) the data analysis is tailored to the stated objective, taking into consideration the categorical nature of the data.

### 2.2 Participants, context, and setting of the multiple case study

The context of the multiple case observation is a Mexican university that conceives learning as significant at the base of the processes of teaching, learning, and assessment processes. The department responsible for teacher training has expressed concern regarding the instructional communication between teachers and students via the offer of training that includes different workshops and a diploma in communicative competence. The institution’s informed idea about the expository class reveals the apprehension about the effectiveness of teaching with this prevalent format at the Master’s Degree level.

The participants in the observed class sessions included 4 teachers and 70 students from urban and semi-urban districts, aged between 23 and 56 (mean 39.5). The teaching staff has an average age of 51.5 (between 40 and 63), is committed to the university education project, and is familiar with instructional communication via basic theoretical training. Furthermore, the lecturers have broad professional teaching trajectories and, in particular, are interested in the effectiveness of teaching about student learning. They are willing to achieve effective encounters.

The work plan was developed in front of the group in an ordinary classroom on a Saturday, with each class session lasting 3 h each. The classrooms are equipped with basic technology, including a computer, projector, and one digital whiteboard. There are between 20 and 30 desks in each classroom—both individual and longer for two or three students—and normal seats or stools. The chalkboard or whiteboard occupies a significant portion of one wall of the classroom, covering various meters, and the arrangement of seats, in general, is traditional.

The observed situations introduced new concepts belonging to two different fields of knowledge within the Master’s Degree. Cases A and D were from two 4-month subjects from the fifth year, theoretical-practical in nature from the Master’s Degree in Mathematical Education. The Mathematical Education lecturers emphasize the value of the expository class with the aim of presenting and developing a great quantity of content progressively in a demonstrative way. In this way, the board and screen play a fundamental role. Cases B and C pertain to two second-year subjects that mainly comprise theoretical material from the Master’s Degree in Pedagogy. The pedagogy lecturers employ new methods of distributing the students in the classroom and activities in an expert expository format. Table 1 summarizes the main heterogeneous characteristics of the four cases^3^.

**Table 1:** Description of the observed situations.

Case	Number of students	Rank of student’s age	Subject type	Academic period	Master
A	9	26–53	Theory Practice	5th	Mathematics
B	26	25–56	Theory	2nd	Pedagogy
C	10	25–40	Theory	2nd	Pedagogy
D	25	23–48	Theory Practice	5th	Mathematics

From a perspective of class session observation, Table 2 synthesizes the aspects of each case: the duration of video recordings, the number of contributions, and frequencies by category.

**Table 2:** Aspects framing the observation in each case.

Case	Duration	Number of contributions	Frequency of category PE	Frequency of category DA
A	2 h. 41′ 11″	871	99	723
B	1 h. 56′	609	41	551
C	2 h. 20′	582	85	490
D	2 h. 7′ 25′	567	89	466
Total	9 h. 6′ 36″	2.629	314	2.230

PE (QUESTION) and DA (GIVE).

**Figure 2:**
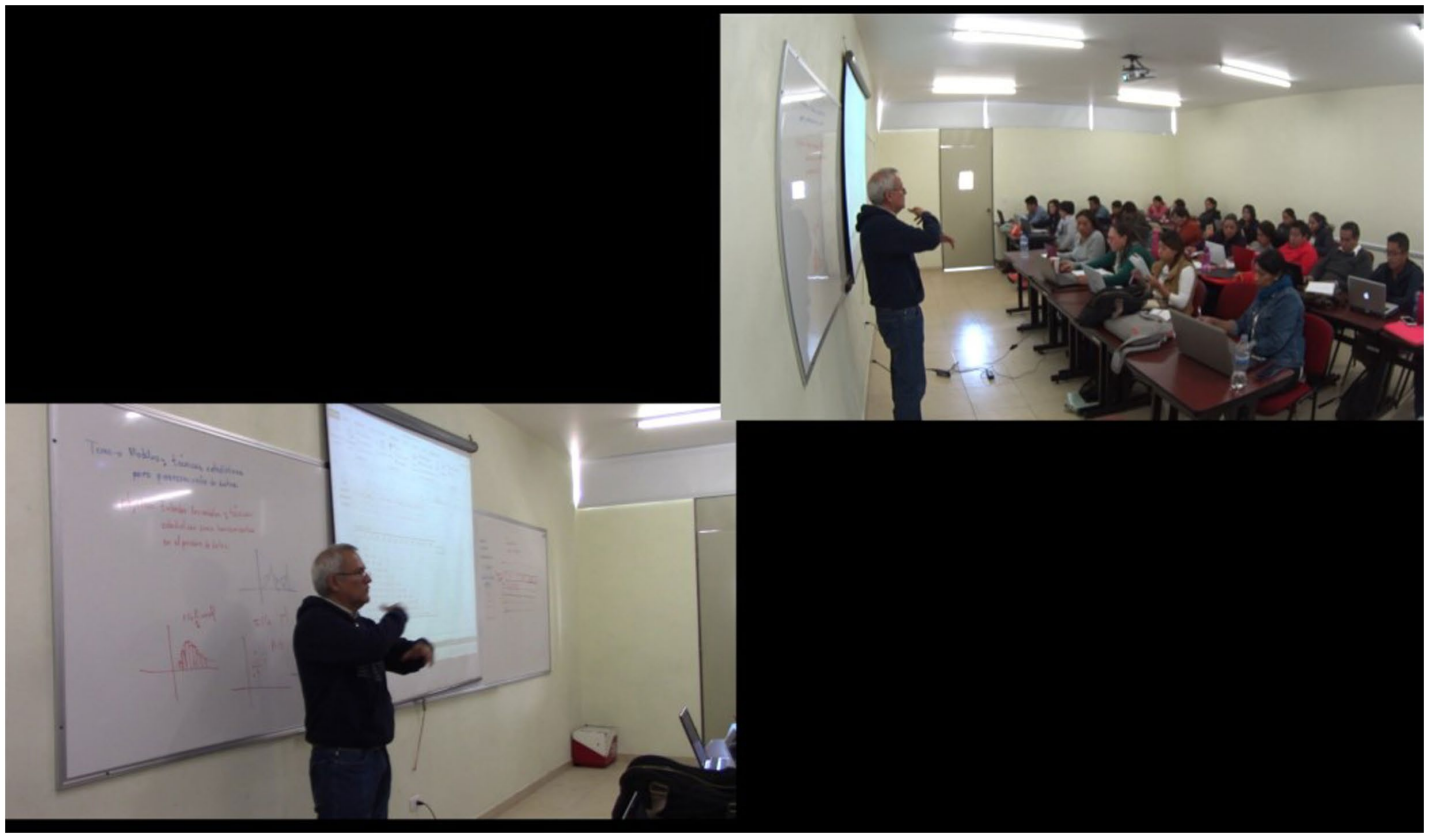
Example of filmed images in Case D.

The recordings required technical material that was installed in each classroom: an omnidirectional microphone, a Sony Action camera with a wide-angle lens, and a Canon camera with a fixed-focus lens. Sound and image editing and montage were carried out by specialized technicians with the Quicktime reproducer and the Final Cut X program, as shown in Figure 2.

### 2.3 Systematic observation instrument

**Figure 3:**
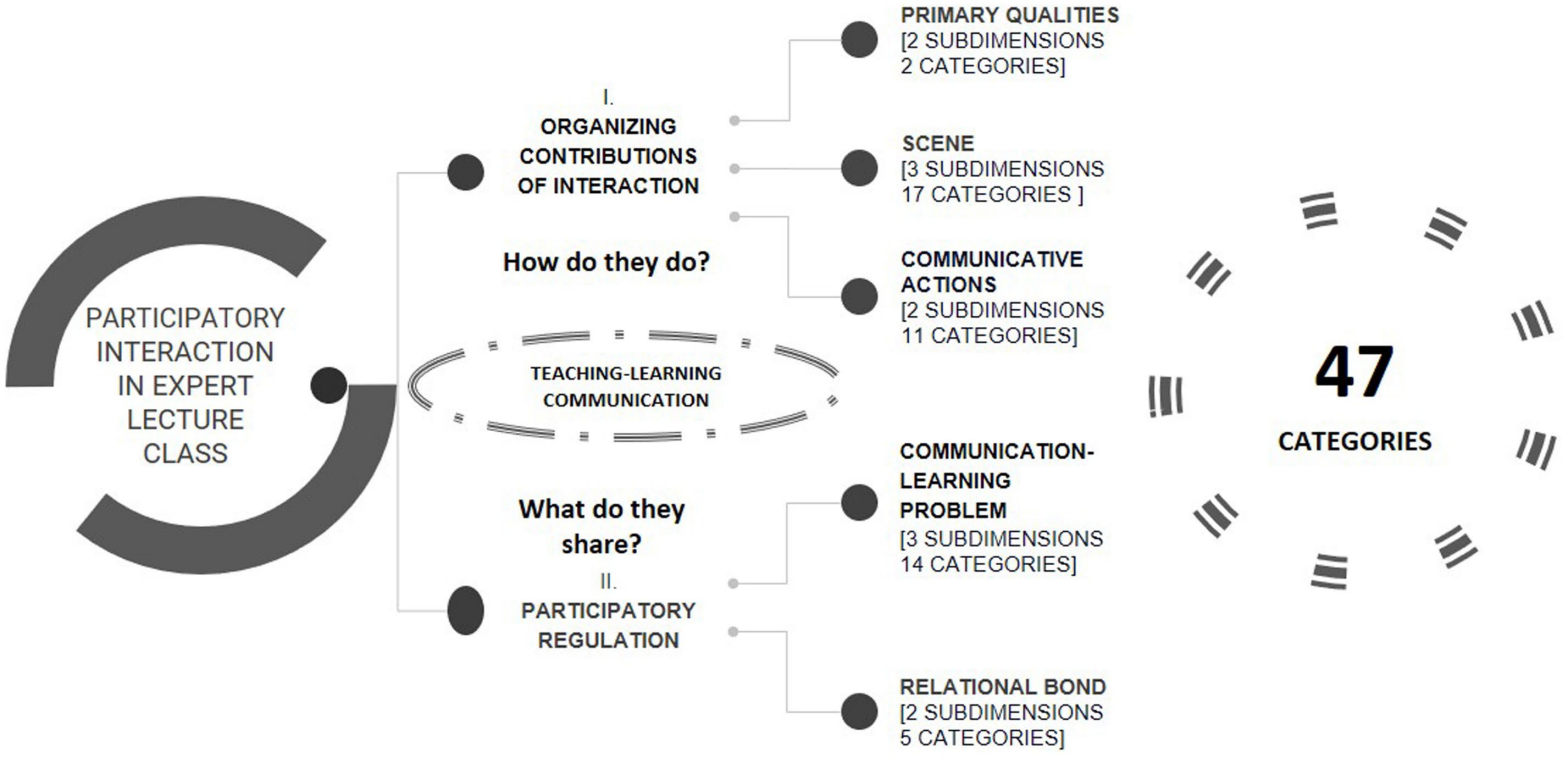
Multidimensional conceptualization and category systems of the systematic observation instrument LUniMex-2017 (Tronchoni et al., 2018a). Own production.

Based on the theoretical conceptualization of the object of study in instructional contexts and in systematic observational methodology (Tronchoni et al., 2018b; Tronchoni, 2019; Tronchoni et al., 2021), the instrument LUniMex-2017 was developed (*Vide*; Figure 3). This approach combines field format with category systems that meet the conditions of exhaustivity and mutual exclusivity for the study of the two macro dimensions: (I) the organizational contributions of the interaction, and (II) the regulation of participation in the construction of knowledge.

### 2.4 Procedure

First of all, authorization to develop the study was sought from the Dean of Social Sciences of the university, and written informed consent was obtained from the lecturers and students in order to carry out the video recordings. The study received the necessary ethical permission for it to be carried out by the institution. The issue of observation was the lecturers’ interactive behavior during a class session with an expository format. The unit of record was the alternating intra- or inter-turn contribution (Duncan and Fiske, 1977), with a minimum duration of 1 s per turn. A passive observation, sampling, transcription, and systematic record of the filmed sessions were executed. Class sessions were coded using the observation instrument LUniMex-2017. The data matrix derives from the systematic record of the categorical coding within the open-access program LINCE (http://observesport.com/; Gabin et al., 2012; Table 3). The concordance calculation and lag sequential analysis were performed using the open-access program GSEQ (https://www.mangold-international.com/en/products/software/gseq.html; Bakeman and Quera, 2011). We then examined the records of the four cases in conjunction with the aggregation of files into 15 groups and through four aggregation levels in order to carry out a lag sequential analysis and detect the possible existence of common patterns PI§EF.

**Table 3:** Illustrative example of the recording and coding processes.

Recording			Coding						
Moment	Duration seconds	Segmentation unit: contribution	Who-to-Whom	Role	Mode	Act	Task	Strategy	Relational bond
0:05–0:07	2	#D: On that date, Freire was also going to be presented, and the colleague who is going to present is on the SEP courses*G	DG	HA	MPR	DA	CI	CEC	IPC
0:08–0:09	1	#G [visual contact]*D	GD	OA	MPR	DA	CI	CEC	IPC
0:10–0:14	4	#D: Although we are going to be lagging behind with the times (…)*G	DG	HA	MPR	DA	CI	UPL	IPC
0:15–0:17	3	#E [directed look]*D	ED	OA	MPR	DA	CI	UPL	IPC
0:18–0:19	2	#D: So they aren’t going to present Freire then, right?*G	DG	HA	MPR	DA	CI	CEC	IPC
0:20–0:21	2	#E [they fiddle with notebooks]*D	ED	OA	MPR	DA	CI	CEC	IPC
0:22–0:25	4	#D: Although chronologically we are going to be jumping around (…)*G	DG	HA	MPR	DA	CI	UPL	IPC
0:26–0:27	2	#E [they smile or laugh]*D	ED	OA	MPR	DA	CI	UPL	IPC
0:28–0:29	2	#D: You already know Freire, so there’s no problem (…)*G	DG	HA	MPR	DA	CI	CCO	IPC
0:30–0:33	4	#E [visual contact or directed look]*D	ED	OA	MPR	DA	CI	CCO	IPC

Codes: DG (Teacher-Group), GD (Group-Teacher), ED (Students-Teacher), HA (Main Speaker), OA (Active Listener), MPR (Proposal-Response Mode), DA (Give), CI (Share Information), CEC (Current Content or Procedures), UPL (Use of the 1st Person Plural), CCO (Knowledge Shared in Class), IPC (Proximal-Warm Exchange).

To guarantee the quality control of the observational records, initially, a consensus agreement was carried out by two researchers possessing extensive knowledge in the subject matter in order to ensure that the content of each category was precisely established and comprehended. During the recording, Cohen’s kappa coefficient (1960) was used to obtain a satisfactory intra-observer agreement of 0.90.

### 2.5 Data analysis

In accordance with the previously outlined observation instrument (*Vide*, Section 2.3), we coded the multiple events observed in each of the episodes that make up the sequential chain of the development of the observed PI§EF sessions.

In order to analyze the transitional probabilities of the multi-event data and verify their significance, we utilized lag sequential analysis (Anguera, 1997; Sackett, 1980). According to the indications of Bakeman and Gottman (1986) and the user instructions for the GSEQ program for the construction of the lag contingency association tables (Bakeman and Quera, 1996, 2011), it is a good idea to limit the number of conditioned behaviors, adjusting them to the aim, and, in addition, to also limit the number of lags. Thus, we have selected 12 categories of interest that have been proposed as conditioned behaviors (Table 4). Each one of these codes belongs to one of the four basic dimensions of the coded observation, and as a given behavior (GB), we focused on the illocutionary action of the QUESTION (*Vide*, Section 1). The given behavior “question” is defined as a verbal and nonverbal message that communicates the need to carry out an action linked to the moment of active comprehension. This is an act of demand or exhortation about something with the possibility of being received or not as answers and replies (ask, ask for, demand, request, invite, etc.).

**Table 4:** Codes used in the lag sequential analysis with Given Behavior and conditioned behaviors.

Dimension SCENE		
Sub-dimensions	Category systems	Codes
Who-to-Whom	Teacher-Group	DG
	Group-Teacher	GD
	Teacher-Students	DE
	Students-Teacher	ED
Dimension communicative acts		
Verbal basicacts	Question (Given Behavior)	PE
	Give	DA
	Show	MO
	Ignore or Reject	IR
Dimension communication-learning problem		
Support strategies to connect with previous knowledge	Previous knowledge of the social framework	CIN
	Knowledge shared in class	CCO
	Individual experience of the social framework	EIN
	Shared experience in class	ECO
Dimension relational bond		
Sociocognitive and emotional regulation	Proximal-Warm Exchange	IPC
	Proximal-Cold Exchange	IPF
	Distant-Warm Exchange	IDC
	Distant-Cold Exchange	IDF

The codes have been maintained according to Spanish nomenclature (Tronchoni et al., 2018b).

In terms of the chaining *Lag* [*L*] of pairs of events (antecedent-criterion [*c*] with subsequent events [*s*]), expressed as p(*s_+L_/c_0_*), it was fixed at five lags (Bakeman and Gottman, 1986). The perspectives analyzed are both prospective (Sackett, 1980) and genuine retrospective (Anguera, 1997).

The rules used to determine when a sequential pattern of behavior conventionally ends were: (a) when there are no more lags with significant behaviors, (b) when there are two consecutive empty lags, and (c) when there are various significant behaviors in two consecutive lags. In this case, the initial of the lags is considered MAX LAG, which marks the interpretative end of the obtained structure (Anguera et al., 2021; Sackett, 1979).

## 3 Results

The results of the file aggregation are presented in table format. The category-given behavior is located in the central column, and the patterns PI§EF are classified in accordance with their structural characteristics: temporal orientation (retrospective/prospective), temporal position of the link of the event conditioned for GB (the five lag positions), nature of the link associative to the event conditioned with GB, and type of conditioned event (positive/*negative*). Each table contains an interpretative description of the patterns found, based on the positive and negative associations identified.

### 3.1 Lag sequential analysis

#### 3.1.1 Aggregation of the files corresponding to ABC, ABD, ACD, and BCD

Twelve significant sequential patterns were selected (*p* < 0.01). We identified four retrospective positives, two retrospective negatives, four prospective positives, and two prospective negatives. Out of 12 sequential patterns, 8 patterns (2 retrospective positive, 2 retrospective negative, 2 prospective positive, and 2 prospective negative) were replicated in the 4 groups of 3, and the 4 remaining were replicated following this distribution: one prospective positive in the case of ABD; one prospective in the cases ABC, ACD, and BCD; one retrospective positive in the case of ABC; and one retrospective positive in the cases ABD, ACD, and BCD (Table 5).

**Table 5:** Classification of PI§EF patterns (*p* < 0.01) for each case: ABC, ABD, ACD, and BCD.

Classification of PI§EF patterns in the ABC Case												
Lag Type	Nature of event	Retrospective					GB	Prospective				
		-R5	-R4	-R3	-R2	-R1	R0	R1	R2	R3	R4	R5
Positive	Global		DG		DG		PE		DG			
	Particular						PE					
	Relational			IPC		IPC	PE	IPC				
	Instrumental	CCO		CCO		CCO	PE	CCO				
*Negative*	*Global*	*DG*	*GD*	*DG*		*DG*	*PE*	*DG*		*DG*		*DG*
	*Particular*				*ED*		*PE*		*ED*		*ED*	
	*Relational*						*PE*					
	*Instrumental*						*PE*					
Classification of PI§EF patterns in the ABD Case												
Positive	Global		DG		DG		PE		DG			
	Particular						PE					
	Relational	IPC		IPC		IPC	PE	IDC				
	Instrumental	ECO		ECO		ECO	PE	CCO				
*Negative*	*Global*	*DG*		*DG*		*DG*	*PE*	*DG*		*DG*		*DG*
	*Particular*		*ED*		*ED*		*PE*		*ED*		*ED*	
	*Relational*						*PE*					
	*Instrumental*						*PE*					
Classification of PI§EF patterns in the ACD Case												
Positive	Global		DG		DG		PE		DG			
	Particular						PE					
	Relational	IPC		IPC		IPC	PE	IPC				
	Instrumental			ECO		ECO	PE	CCO				
*Negative*	*Global*	*DG*		*DG*		*DG*	*PE*	*DG*		*DG*		*DG*
	*Particular*		*ED*		*ED*		*PE*		*ED*		*ED*	
	*Relational*						*PE*					
	*Instrumental*						*PE*					
Classification of PI§EF patterns in the BCD Case												
Positive	Global		DG		DG		PE		DG			
	Particular						PE					
	Relational	IPC		IPC		IPC	PE	IPC				
	Instrumental			ECO		ECO	PE	CCO				
*Negative*	*Global*	*DG*		*DG*		*DG*	*PE*	*DG*		*DG*		*DG*
	*Particular*		*ED*		*ED*		*PE*		*ED*		*ED*	
	*Relational*						*PE*					
	*Instrumental*						*PE*					

Positive sequential associations are developed in exchanges that mobilize the global attention of the class group and relational and instrumental strategies (prospective) in the four cases. There exists a possibility of being able to observe the illocutionary PE action subsequent to having observed a global communicative moment and relational and instrumental strategies (retrospective).

Negative sequential associations are the values “global/particular” of the speech direction event that were observed much fewer times than expected by chance in alternate positions for each value.

#### 3.1.2 Aggregation of files corresponding to ABCD

The search for replicated patterns culminated with the aggregation of the data obtained in A, B, C, and D. Once again, 12 significant sequential patterns were obtained: 3 retrospective positive, 3 retrospective negative, 3 prospective positive, and 3 prospective negative (Table 6).

**Table 6:** Classification of the PI§EF patterns (*p* < 0.01) in the ABCD file aggregation.

Lag type nature of event		Retrospective					GB	Prospective				
		-R5	-R4	-R3	-R2	-R1	R0	R1	R2	R3	R4	R5
Positive	Global		DG		DG		PE		DG			
	Particular						PE					
	Relational	IPC		IPC		IPC	PE	IPC				
	Instrumental			ECO		ECO	PE	CCO				
*Negative*	*Global*	*DG*		*DG*		*DG*	PE	*DG*		*DG*		*DG*
	*Particular*		*ED*		*ED*		PE		*ED*		*ED*	
	*Relational*						PE					
	*Instrumental*		*CIN*		*CIN*		PE		*CIN*		*CIN*	

Positive sequential associations: the action “question” is developed in exchanges that mobilize the global attention of the class group, and relational and instrumental strategies (prospective) in the four cases. There exists the possibility of observing the illocutionary PE action in R0 when lag-positive associations have previously been observed before (-R1 a -R5) between PE and DG, PE and IPC, and PE and CCO.

Negative sequential associations: the values “global/particular” of the speech direction event were observed much fewer times than expected by chance in alternate positions for each value. The same thing happens with the “prior knowledge” instrumental strategy of the social framework.

### 3.2 Theoretical interpretation of the replicated patterns in the aggregation of files ABCD

If the social and scientific commitment derived from the analysis is to establish the level of intervention understood as a co-constructive method, it is imperative to summarize—without omitting any pertinent information—the adjustment model found among the most replicated patterns (*Vide*; Table 4) shared by the four observed cases (*Vide*; Table 5), within the restriction of limiting: (a) the associations conditioned by PE to the positions -R2 or -R1 and +R1 or +R2, (b) the relationships of inhibition to those values that did not obtain retrospective or prospective positive presence in other lagged positions, and (c) the unnecessary repetition of events.

The adjustment issuer-receiver/expert-learner is related to different categories of instrumental and relational content that, in order to be received or taken with a view to being negotiated and shared at different comprehension levels, need to be provided in accordance with the discursive moment of the exchanges and their combinations: establish bridges, give meaning, and elaborate relationship networks (Tronchoni et al., 2018b).

**Figure 4:**
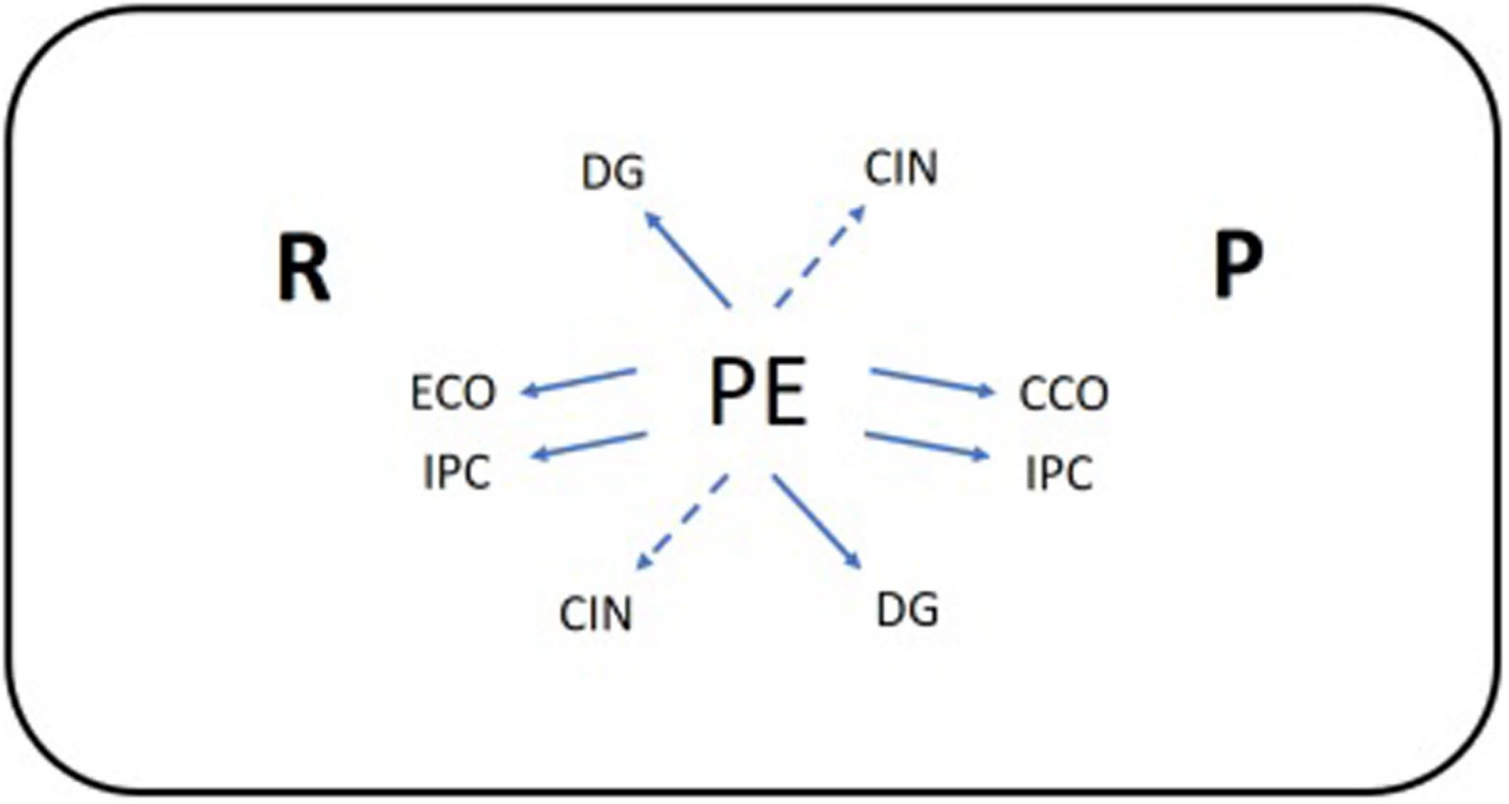
Conceptual-interpretative scheme of adjustment model about experience and knowledge sharing in the classroom: PE ECCOipc§EF. Symbols: prospective (P), retrospective (R), positive association (continuous arrow); negative association (dashed arrow). Own production.

The discoveries from the aggregations of the files ABC, ACD, and BCD coincide in incorporating the prospective pattern PE IPC, and the retrospective pattern PE ECO forms part of the cases ABD, ACD, and BCD. The prospective pattern PE CCO is shared in the four cases. On examining the sequential patterns, taking into account the aforementioned restrictive rules, we discover (Figure 4):

- The reflectivity-continuity before/after a mode of IPC relationship that guides the CCO exchanges.
- The prospective positive association of PE with CCO which provides the IPC relationship with instrumental content.
- The retrospective positive association of PE with ECO which provides the IPC relationship with instrumental content.
- The global preceding-consequence DG contribution is positively associated with the category PE.
- The diminution or blocking of both the retrospective and prospective negative association of *CIN* PE *CIN*.

## 4 Discussion and conclusions

The evaluative-formative objective of this research into instructional communication situates the discussion of the presented outcomes with the realm of educational intervention. In line with a relational vision of the communication that takes place via languages and psychosocial relationships, the interactive process has been designed to recognize the speakers’ participation, i.e., both extremes of the interaction listen and transact (Anguera and Izquierdo, 2006; Izquierdo, 1996; Izquierdo and Anguera, 2021; Izquierdo and Perinat, 2010; Watzlawick and Jackson, 2010; Winkin, 1981).

The principles of communication (Grice, 1975; Marc and Picard, 1989; van Dijk and Kintsch, 1983; Watzlawick and Weakland, 1977) that support the instrument of systematic observation, LUniMex-2017, are (Tronchoni et al., 2018b): (a) the concept of feedback and its various functions, (b) the dialogism inherent in verbal interaction, (c) the situation and the activity’s objectives, (d) the exchange of meanings, (e) the multimodal construction of meaning, (f) the reciprocity or recognition of the other as a valid interlocutor, and (g) mutual influence.

The reading of the PI§EF process was contextualized from the educational viewpoint in the principles of social constructivism, which structure the educational discourse (Coll, 1996; Coll and Onrubia, 2001; Coll et al., 1992; García-Fariña et al., 2018; Prados and Cubero, 2005; Rogoff, 1990) around the teacher-student shared task of adjusting the expert explanation and the activity of active-comprehending listening as a process of guided and conscious dialogical inquiry (Ur, 1994; Fernández and Cuadrado, 2008).

The detection by aggregation of cases (Anguera, 2018) of shared significant patterns in the analyzed combinations of triples and quaternary (see other examples, Alarcón-Espinoza et al., 2024; García-Fariña et al., 2018) has enabled us to concentrate the descriptive return of the observed instructional behavior into a theoretical-interpretative diagram showing the complexity of the exchanges motivated by the illocutionary action “question” (PE). The educational assistance identified in the significant and replicated link between PE and the categories “speech direction” and “exchange orientation” includes: (a) regulatory dialogical sequences of how we communicate and what we share, (b) dialogical sequences of attention opening and subject advancement, and (c) dialogical sequences of closure with significance.

**Figure 5:**
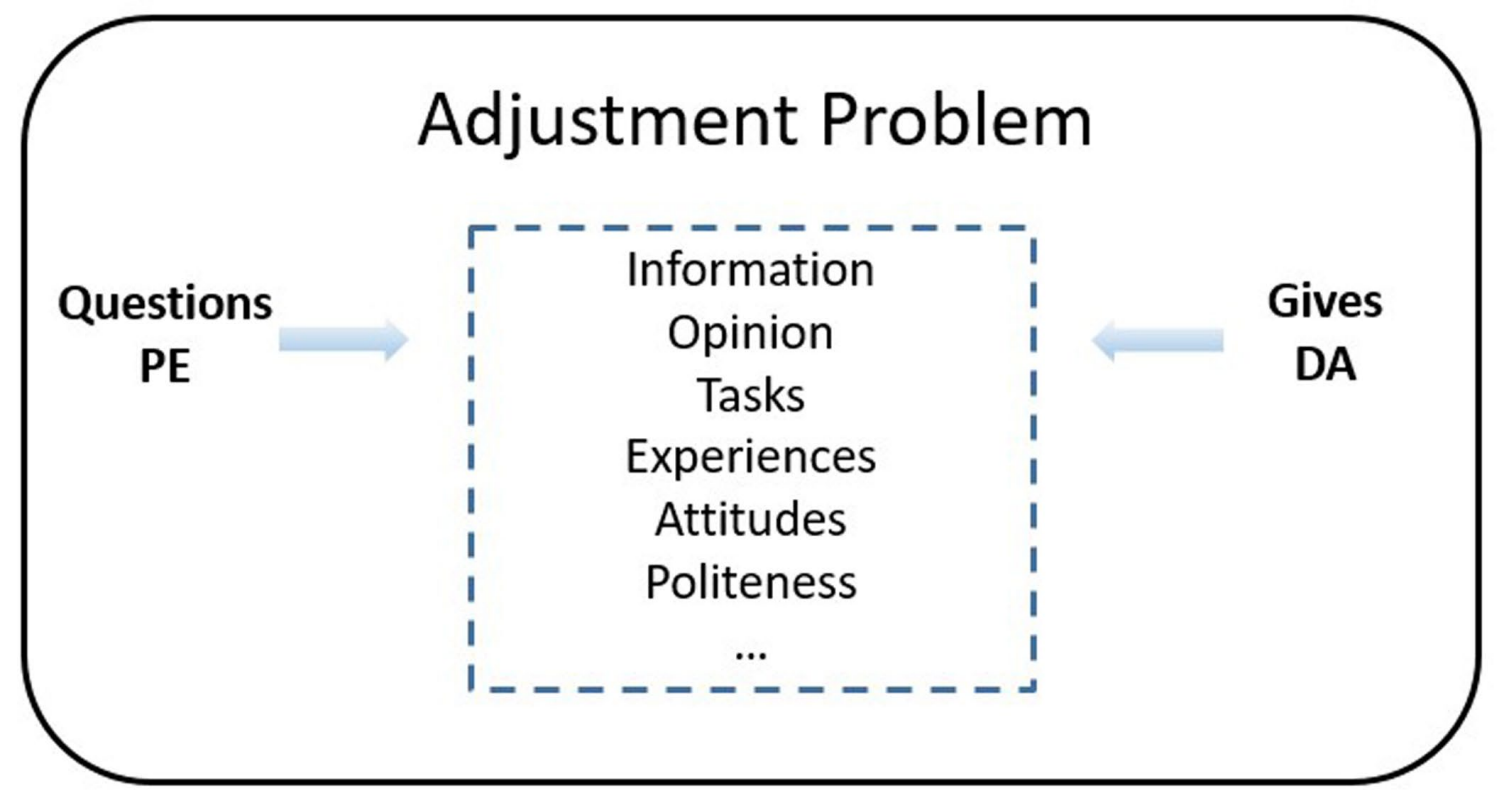
Adjustment problem between the communicative acts *Questions* and *Gives*. Own production.

The communicative mode PE is further complemented by the communicative mode “give” (DA). Both modes of communication attempt to identify both instrumental and relational issues of adjustment between the attending students and the expert teacher (Figure 5).

We comprehend that these two distinct movements of educational support pertaining to language are generated in different ways. Maintenance of the fluid deployment of the sequential or planned educational support in PE mode is fed by the lecturer’s (D) search for information about the course of the interaction to assess what questions to ask in order to reinforce and channel the students’ active-comprehending listening (E). However, when the teacher executes action DA, the search for information is centered on the evaluative behavior displayed by the students (E) minute by minute throughout the explanation. It is noteworthy that the critical position of D in DA is not altered by educational support; it does not modify the course of the instructional communication dialogue until the lecturer incorporates the listeners’ message received in DA.

The instructional intervention, supported by a systematic observation study and envisaged as a constructivist learning environment via the viewing of the produced digital videos, involves (Jonassen and Rohrer-Murphy, 1999) returning the results incorporated into the theoretical-interpretative diagram, exposing the filming to dialogued self-observation, and establishing a plan of improvement. The use of video analysis in education and in sports training (Chicote et al., 2009; Cushion, 2006; Hernández-Mendo and Anguera, 2000; Lemyre et al., 2007) provides evidence about the benefits it can bring. In our case, it is worth mentioning that self-observation and the discussion of the video recordings connect with the expectations and interests of the teachers involved.

Four examples are presented, one for each case, interpreted according to the theoretical interpretative diagram of the adjustment model PE ECCOipc§EF (*Vide*; Figure 4). The examples are accompanied by: (i) the adjustment model of experience and shared knowledge in class (ECCO) based on feedback, (ii) the scaffolding mode—sequential or planned, and (iii) a descriptive framework that places each example in the temporal phase of the class session (start, middle, or end), the didactic moment it corresponds to (introduction, development, or closure) and the subject, followed by the subject.

**Table:** 

Case A: Adjustment model of experience and shared knowledge in class which describes an accumulative confirmatory feedback of work rules or regulations. Planned scaffolding. The teacher is a lecturer, the example takes place during the final temporal phase of the class session, the didactic moment is of closure and the subject Mathematics of Change. Student: *Excuse me, err… when do we have to hand in the portfolios?* Teacher: *I suggest that if we don’t see each other again in eight days time, that there is some activity online* (…) *I propose that you hand it in by the following week, we agreed that it would in a Word file, didn’t we*? [PE: Support requirement] Student: *Yes* Teacher: *I think that would be the last week, I will be opening a link in the first few days.* Case B: Adjustment model of experience and shared knowledge in class which describes an accumulative dynamic feedback. Planned scaffolding. The teacher is a lecturer, the example takes place during the initial temporal phase, the didactic moment corresponds to the introduction and the subject is Educational Currents. Teacher: *We have to discuss the Renaissance. Last week we talked about the Middle Ages, how it starts and how it ends, the importance of monastic groups (…), how universities come into being there, right?* [PE: Invitation to the subject] Student: (*the students are in preparation mode, taking things out of their bags and placing them on the tables; visual contact is being initiated between the students and the lecturer.)* Case C: Adjustment model of experience and shared knowledge in class which describes a feedback of correct reception. Sequential scaffolding. The teacher is a lecturer, the example takes place during the initial temporal phase, the didactic moment corresponds to the introduction and the subject is the Foundations of Educational Orientation. Teacher: *And I was saying that it was associated to which type of model? Do you remember? (pause) From the models we reviewed at the beginning. (pause) (the lecturer is sitting in the oval formed by the distribution of the group, her gaze sweeps around the group of students and her tone of voice is linear with confirmation modulations: aha / right?)* Student: *To the consulting* [model] Teacher: *To the consulting and clinical* [model]*, right?* [PE: Joint negotiation-action] Case D: Adjustment model of experience and shared knowledge in class which describes a dynamic-sequential/planned feedback. Sequential scaffolding. The teacher is a lecturer, the example takes place in the initial temporal phase, the didactic moment is that of development, and the subject is Information Analysis Processes. Teacher: *What is statistical correlation?* (*…*) *(gaze directed towards the PPT alternating with directionality towards the group at the front of the class) Do you know why? Because it could be a rule of mathematical correspondence* (*…*) *we can do a (inaudible) geometric, draw it, either in second or third dimension.* [PE: Joint negotiation-action] Student: *(two students join the class session; others are taking notes on their laptops and in their notebooks)* Teacher: *(addressing the group) We had already seen it too. When I have an experiment that gives me many points (writing on the board)*

These examples of the adjustment model PE ECCOipc§EF are reflected in the different realities lived and experienced by the four lecturers and embody the interpretative diagram based on the knowledge adjustment shared in class and the analysis of the multiple cases. Therefore, within the framework of participatory action research (*Vide*, Section 1), the professional educational task requires the teacher to self-observe via the viewing of filmed sessions, moving backward and forward, and stopping in exemplifying situations to describe feedback management and the sequential and planned structure of PI§EF.

The instance of Case A takes place within a scenario that seeks the confirmation (*when do we have to hand in the portfolios?*) of established rules regarding the study and autonomous development of the work. The situation in Case B corresponds to a dynamic feedback that connects and mobilizes the thematic plan of the course (*Last week we talked about the Middle Ages*), and it is anticipated that active listening will be established (*the students are in preparation mode, taking out material*) on what follows and commences the session. Both accumulative examples, owing to their sedimentation of the ongoing work, correspond to a planned scaffolding that guides the conduct of the exposition.

Case C is within a scenario that seeks to ensure the correct reception (*to the consulting and clinical* [model], *right?*) of thematic content (models of educational orientation) studied prior to the filmed class session. The situation in the Case D example corresponds to revitalizing feedback mobilized through the act of questioning (*Do you know why?*), in which the lecturer knows the difficulties the students might have in the action of listening-comprehending, reducing the cognitive distance (*because it could be a rule of mathematical correspondence*). Both examples—Cases C and D—correspond to a sequential structure plan derived from an *in situ* interactive sequence between the lecturer and the students.

In terms of the four examples provided here, there are some advanced guidelines to assist the teacher to address basic and important issues, such as: the need to plan autonomous work and study together with the students (Case A); the importance of preparing the necessary resources that affect attention from the beginning of the session, and adopting WE BEGIN mode (Case B); to strengthen objectivism about the circumstances, conditions, and consequences related to learning consolidation (Case C); and mediate cognitive distance with the incorporation of longer pauses following a question that permit the students to establish connections between their prior knowledge through oral contributions (Case D).

The work of teacher self-observation is guided by the discussion of the results from the practiced systematic observation and used as a support of the lived experience in viewing the filmed sessions. This training exercise process involves the conscious, deep, and reflexive vision of the teacher, channeled toward a plan of improvement of what is being done and how it is being done in terms of educational support. As previously stated in the introduction, the objective of PI§EF is to generate and cultivate the expertise of the listener as a communicative actor and as a learner of academic content.

In light of the above, it can be concluded that there exist two key moments in the pursuit of improving the preparation of the expert expository class. These are the updated, substantial, and well-documented moments of assembling the expository plan of curricular content and the moment of preparing communicative interaction, i.e., how to communicate it in the face of the communication-learning problem (*Vide*; Figure 2), via mechanisms of feedback and the discursive strategies on which the asymmetric commitment of the actors relies.

By way of conclusion, the above examples of the PE ECCOipc§EF adjustment model demonstrate the instructive potential of revealing what was previously hidden from the participants because they did not have the conceptual and analytical tools to objectively deal with the complexity of PI§EF. The four examples form a narrative of the captured images. The results of the observational research conducted are presented to the professional forum responsible for assessing the efficacy of the instructive proposal based on video analysis, backed by a previous systematic observation study conducted within the framework of the mixed methods approach with a multi-case study.

The limitations of this study are twofold: first, it is imperative to augment the number of trained observers; second, it is imperative to adhere to the training and consensus guidelines of the work and evaluate the agreement between observers until the application of the statistics specific to observational methodology. However, the instrument is clear and transparent and therefore has great potential for communication with the scientific and professional community. Furthermore, the laborious work of delimiting, naming, and defining the categories means that it is adaptable to different educational contexts. Furthermore, it allows us to progress through the molecularization of category systems in the morphology of comprehensive attention of the interpretative parts of the expository class, for the benefit of teacher training.

## Data Availability

The raw data supporting the conclusions of this article will be made available by the authors, without undue reservation.

## Footnotes

^1^From now on we will use “question” to refer to ask for, ask questions, request, demand, invite, etc.

^2^§ specific rules for implementing the expert expository format (EF).

^3^For more information about the participants, the context, and the scene, consult the following references: Tronchoni, 2019; Tronchoni et al., 2021.
