# Supplementary material for: Systematic observation of participatory interaction in university lectures: a multiple case study with a mixed methods approach
Source: Front Psychol. 2024 Nov 1;15:1410486. doi: 10.3389/fpsyg.2024.1410486 (PMC11565398; doi:10.3389/fpsyg.2024.1410486)
Supplement: Supplementary file 1 [file Table_1.docx]

**Observational Instrument LUniMex-2017**

Based on the theoretical conceptualization of the object of study in instructional contexts and systematic observational methodology, the Instrument LUniMex-2017 is structured with 2 macro dimensions, 5 dimensions, 12 subdimensions, and 47 categories. This combines field format with category systems that meet the conditions of exhaustivity and mutual exclusivity for the study of the two macro dimensions: I) the organizational contributions of the interaction, and II) the regulation of participation in the construction of knowledge.

This table shows dimensions, subdimensions, category systems, definitions, and codes, and it is adapted from Tronchoni (2019) and Tronchoni et al. (2018b, 2021).

| **MACRO DIMENSION I:**  **ORGANIZING CONTRIBUTIONS TO THE INTERACTION** | | | |
| --- | --- | --- | --- |
| Dimensions | Subdimensions and Category systems | Definitions | Codes |
| Primary qualities | Sequential order |  | [000] |
|  | Time |  | [hh:mm:ss] |
|  | Exchange duration in seconds |  | [00:00] |
| Scene | Who-to-whom | Emitter/receiver and receiver/emitter dyads | QaQ |
|  | Teacher-Group  Group-Teacher  Teacher-Students  Students-Teacher  Teacher-Male Student  Male Student-Teacher  Teacher-Female Student  Female Student-Teacher | Teacher addresses the group  Group addresses the teacher  Teacher addresses the students  Students address the teacher  Teacher addresses a male student  Male student addresses the teacher  Teacher addresses a female student  Female student addresses the teacher | DG  GD  DE  ED  DEo  EoD  DEa  EaD |
|  | Role in the speaker’s use of the word | Turn position from an oral contribution | RUP |
|  | Main speaker  Secondary speaker  Active listener  Listener (instrumental) | Teacher's oral contributions  Participant's oral contributions when she/he/they are not the main speaker  or when the teacher has a speaking secondary position  Backchannel positive feedback contributions  Oral contributions with a manifested absence of backchannel behavior | HA  HI  OA  O |
|  | Exchange mode | Way in which participants perceive the relation between an oral contribution and other contributions | MIN |
|  | Proposal-response mode  Positive self-replication  Negative self-replication  Positive alter-replication  Negative alter-replication | Proposal contribution is followed by a response contribution  Positive feedback value is assigned to the self-speaking contribution  Negative feedback value is assigned to the self-speaking contribution  Positive feedback value is assigned to the other-speaking contribution  Negative feedback value is assigned to the other-speaking contribution | MPR  URPP  URPN  ARPN  ARPN |
| Communicative acts of the participants | Basic verbal acts | Communicative acts that are present in the speaking exchange | ABA |
|  | Question  Give  Show  Ignore or Reject | Demand or exhortation about something  Offering, delivery, or cessation of something  Indication or sample of something  Communicative act is ignored or rejected | PE  DA  MO  IG |
|  | Emitter-receiver adjustment task | Interactive content that the emitter-destinator asks, gives, or shows | TAJ |
|  | Share information  Share opinion  Share homework  Share instruction  Share experience  Share attitude  Share courtesy | Oral contribution has data, events, figures, and information about people, content, and situations  Oral contribution includes opinions, beliefs, comparisons about people, content, or situations  Oral contribution refers to activities or tasks to do  Oral contribution includes guidelines, rules, and orders affecting the expository format  Oral contribution focuses on the interest to express and share experience  Oral contribution has ratings and feelings about the expository content  Oral contribution includes directions or manners about respectful interpersonal treatment | CI  CO  CD  CN  CE  CA  CC |

| **MACRODIMENSION II:**  **REGULATION OF PARTICIPATION IN THE CONSTRUCTION OF KNOWLEDGE** | | | |
| --- | --- | --- | --- |
| Dimensions | Subdimensions and Category systems | Definitions | Codes |
| Communication  learning problem | Support strategies that control the objective of establishing bridges | Teacher or student's contributions to sharing the interactive content | EEP |
|  | Previous knowledge of the social framework  Knowledge shared in class  Individual experience of the social framework  Shared experience in class | Individual ideas, concepts, or procedures, acquired out of the course context, about content to study  Collective ideas, concepts, or procedures about content studied previously in class  Previous situation (achievements, obstacles, or concerns) about contents to study  Previous situation (achievements, obstacles, or concerns) about content studied previously in class | CIN  CCO  EIN  ECO |
|  | Support strategies that control the objective of attributing meaning to the master class | Teacher or student's contributions to engage the non-expert in the expository format | EAS |
|  | Current content or procedures  Use of the 1st person plural  Question followed by a response  Incorporation of contributions | Ideas, concepts, and procedures during the sessions  Use of verbs or pronouns in 1st plural persona, explicitly or implicitly  Questions followed by the answer about the content or procedures in progress  Incorporation (word by word, paraphrasing, or reformulating) of other participants' contribution | CEC  UPL  PRE  ICO |
|  | Support strategies controlling the objective of advancing the content elaboration process of the exhibition | Teacher or student's contributions to test the comprehension progress | ELA |
|  | Recapitulation  Summary  Synthesis  Categorization or labeling  Re-elaboration  Change of referential perspective | Mention of activities done  Mention the content studied  Mention the relation between activities or contents with previous ones  Labeling elements from content, context, or activity  Participants' contributions reorganize or nuance an idea  Draw the attention of the listeners by referring to elements from the content, context, or activity | REC  RES  SIN  CAT  REE  CAM |
| Relational bond | Socio-cognitive proximity (presence) / distance (absence) regulation and emotional warm (presence) / cold (absence) | Interpersonal immediacy of the instrumental or socioemotional exchanges about  the presence or absence of proximity-distance and warm-cold dyads | RPS/RCE |
|  | Proximal-Warm Exchange  Proximal-Cold Exchange  Distant-Warm Exchange  Distant-Cold Exchange  Neutral Exchange | Behavior refers to the presence of a) the emitter-destinator's direct support help/feedback and b) emotional immediacy-expressivity through the destinator-receiver receives the relaxed and rewarding mode (familiar mode)  Behavior refers to a) the presence of the emitter-destinator's direct support help/feedback and b) the absence of emotional immediacy-expressivity through the destinator-receiver receives the relaxed and rewarding mode (academic standard mode)  Behavior refers to a) the absence of the emitter-destinator's direct support help/feedback and b) the presence of emotional immediacy-expressivity through the destinator-receiver receives the relaxed and rewarding mode (familiar mode)  Behavior refers to the absence of a) the emitter-destinator's direct support help/feedback and b) emotional immediacy-expressivity through the destinator-receiver receives the relaxed and rewarding mode (academic standard mode)  No classifiable relation according to the previous categories | IPC  IPF  IDC  IDF  INE |
